# Nitric Oxide System and Bronchial Epithelium: More Than a Barrier

**DOI:** 10.3389/fphys.2021.687381

**Published:** 2021-06-30

**Authors:** María Amparo Bayarri, Javier Milara, Cristina Estornut, Julio Cortijo

**Affiliations:** ^1^Department of Pharmacology, Faculty of Medicine, University of Valencia, Valencia, Spain; ^2^Biomedical Research Networking Centre on Respiratory Diseases (CIBERES), Health Institute Carlos III, Madrid, Spain; ^3^Pharmacy Unit, University General Hospital Consortium of Valencia, Valencia, Spain; ^4^Research and Teaching Unit, University General Hospital Consortium of Valencia, Valencia, Spain

**Keywords:** bronchial epithelium, nitric oxide, nitric oxide synthase, soluble guanylyl cyclase, cyclic guanosine-3′, 5′-monophosphate

## Abstract

Airway epithelium forms a physical barrier that protects the lung from the entrance of inhaled allergens, irritants, or microorganisms. This epithelial structure is maintained by tight junctions, adherens junctions and desmosomes that prevent the diffusion of soluble mediators or proteins between apical and basolateral cell surfaces. This apical junctional complex also participates in several signaling pathways involved in gene expression, cell proliferation and cell differentiation. In addition, the airway epithelium can produce chemokines and cytokines that trigger the activation of the immune response. Disruption of this complex by some inflammatory, profibrotic, and carcinogens agents can provoke epithelial barrier dysfunction that not only contributes to an increase of viral and bacterial infection, but also alters the normal function of epithelial cells provoking several lung diseases such as asthma, chronic obstructive pulmonary disease (COPD), cystic fibrosis (CF) or lung cancer, among others. While nitric oxide (NO) molecular pathway has been linked with endothelial function, less is known about the role of the NO system on the bronchial epithelium and airway epithelial cells function in physiological and different pathologic scenarios. Several data indicate that the fraction of exhaled nitric oxide (F_E_NO) is altered in lung diseases such as asthma, COPD, lung fibrosis, and cancer among others, and that reactive oxygen species mediate uncoupling NO to promote the increase of peroxynitrite levels, thus inducing bronchial epithelial barrier dysfunction. Furthermore, iNOS and the intracellular pathway sGC-cGMP-PKG are dysregulated in bronchial epithelial cells from patients with lung inflammation, fibrosis, and malignancies which represents an attractive drug molecular target. In this review we describe in detail current knowledge of the effect of NOS-NO-GC-cGMP-PKG pathway activation and disruption in bronchial epithelial cells barrier integrity and its contribution in different lung diseases, focusing on bronchial epithelial cell permeability, inflammation, transformation, migration, apoptosis/necrosis, and proliferation, as well as the specific NO molecular pathways involved.

## Introduction

Bronchial epithelium is directly in contact with the environment and thus, its barrier function is essential to protect the lung from the entrance of pathogens, allergens, or irritant particulates and to maintain homeostasis (Bals and Hiemstra, 2004; Whitsett, 2015). The principal components that maintain the barrier function of airway epithelium are the tight and adherens junctions, the mucociliary clearance, and the antimicrobial products secretion (Ganesan et al., 2013).

On the apico-lateral border of epithelial cells are present tight junctions, adherens junctions, and desmosomes forming the apical junctional complex (AJC). These proteins are connected to the cytoskeleton and fundamental to maintain the structure of the airway epithelium (Whitsett, 2015). The proteins that form the tight junctions such as occludin, claudin family, junctional adhesion molecule (JAM), and zonula occludens (ZO) are linked to the actin cytoskeleton and regulate paracellular transport of ions and some molecules. Meanwhile, the proteins involved in the formation of adherens junctions, such as E-cadherin, are also linked to the actin cytoskeleton and are essential for cell-cell adhesion and intracellular signaling (Rezaee and Georas, 2014; Rusu and Georgiou, 2020). E-cadherin regulates several cellular processes mainly through the binding and sequestration of β-catenin. The formation of this complex avoids the translocation of β-catenin into the nucleus regulating pathways involved in proliferation, cell recognition, polarization, and cell migration, among others (Wong et al., 2018; Rusu and Georgiou, 2020). On the other hand, desmosomes are linked to the intermediate filament cytoskeleton and are also important in intercellular junctions giving mechanical strength to tissues (Garrod and Chidgey, 2008). Thereby, the AJC complex regulates the epithelium permeability by avoiding the entrance of inhaled pathogens and environment particulates and preventing the diffusion of soluble mediators or proteins between apical and basolateral cell surfaces. Furthermore, the AJC participates in several signaling processes of genic expression, differentiation, apoptosis, cellular proliferation, and immunological responses (Balda and Matter, 2009; Inoue et al., 2020).

The mucociliary clearance also prevents the entry of pathogens or particles into the lung. The mucus traps these microbes or particles, and the beating of ciliated epithelial cells carries them forward to the pharynx (Ganesan et al., 2013).

Airway epithelial cells also secrete several molecules, proteins, and peptides such as enzymes, protease inhibitors or oxidants that accumulate in the airways surface liquid and regulate inflammation, chemotaxis, antimicrobial defense, antioxidant levels, repair, and remodeling (Gohy et al., 2020). These functions are key important to avoiding the entry of pathogens and harmful particles without inducing inflammation.

Apart from the barrier function, the bronchial epithelium also can modulate the immune response and integrate all the pulmonary defenses. Allergens may contain proteases that directly damage the airway epithelium and the AJC complex. These proteases are recognized by epithelial protease-activated receptors (PARs) and trigger the activation of the immune response (Ramu et al., 2018). Additionally, epithelial cells widely express pattern-recognition receptors (PRRs), such as NOD-like receptors and Toll-like receptors (TLRs), which recognize and respond to pathogen-associated molecular patterns (PAMPs) and danger-associated molecular patterns (DAMPs). Thus, in response to these stimuli, epithelial cells produce cytokines, chemokines, growth factors, lipid mediators, and DAMPs to interact with themselves and to recruit and activate effector cells and antigen-presenting cells (Davies, 2014; Whitsett, 2015).

Therefore, bronchial epithelium plays a key role in maintaining pulmonary homeostasis. Disruption of one or more of the epithelium functions by harmful particles or pathogens causes epithelial barrier dysfunction, which entails an increase of epithelial permeability and susceptibility to infection, and often an exaggerated long-term inflammation that contributes to various chronic lung diseases such as chronic obstructive pulmonary disease (COPD), asthma (Xiao et al., 2011; Ganesan et al., 2013) or cystic fibrosis (CF) (Cabrini et al., 2020) between others. Dysregulation of intercellular adhesions and cell polarity with a loss of epithelial integrity have been observed also in patients with lung cancer (Bonastre et al., 2016).

Between molecules secreted by the epithelial cells, the nitric oxide (NO) is a lipophilic gaseous transmitter involved in a wide number of signaling and regulation physiological processes in which is included vasodilation, smooth muscle relaxation, neurotransmission regulation, and different inflammatory processes like platelet aggregation inhibition (Moncada et al., 1997; Szabo, 2010; Zou et al., 2020). In the respiratory epithelium, NO is also a key regulator of several airway epithelial physiological functions. Among them would be remarkable his role in the mucociliary function and ciliary frequency (Li et al., 2000), in the epithelial ion transport (Hardiman et al., 2004), in barrier dysfunction restoration after injury by wound repair processes (Olson et al., 2009), and in the modulation of inflammation by regulation of epithelial production of inflammatory mediators, contributing to innate host defense (Bogdan, 2001). In the present review, the role of NO on the bronchial epithelial barrier integrity and its relationship with lung diseases will be discussed.

### NITRIC OXIDE (NO) GENERATION AND NO SYNTHASES

From the amino acid L-arginine, in a reaction oxygen- and NADPH dependent, the NO synthases family (NOS) produces NO and L-citrulline (van den Berg et al., 2018). It is possible to differentiate three NO synthase isoforms, the neuronal NOS (nNOS or NOS1), the inducible NOS (iNOS or NOS2) and the endothelial NOS (eNOS or NOS3). nNOS and eNOS are considered constitutive NO synthases and their activation are dependent on intracellular calcium concentration. Conversely, iNOS is particularly expressed in epithelial cells and macrophages in response to cytokines and/or proinflammatory stimuli, and it produces NO independently of calcium concentration (Moncada et al., 1997; van den Berg et al., 2018). However, in some circumstances, nNOS and eNOS expression may be inducible, and iNOS expression constitutive. Specifically, in the lung epithelium, there is a constitutive iNOS expression. This might be because NO is essential to maintain barrier integrity, avoid the entrance of pathogens, and regulate ciliary beating, among other functions, processes that will be more detailed below (Mattila and Thomas, 2014).

iNOS expression is mostly regulated at the transcriptional level although there are also translational, and posttranslational mechanisms involved in iNOS expression and function. The iNOS gene promoter is very complex and is activated by an additive effect of various transcription factors such as AP-1, C/EBP, CREB, GATA, HIF, IRF-1, NF-AT, NF-κB, NF-IL6, Oct-1, PARP1, PEA3, p53, Sp1, SRF, STAT-1α, TBE, TCF, and YY1 (Pautz et al., 2010; Guo et al., 2016). In epithelial cells, the combined action of some cytokines, being the most important interleukin 1β (IL-1β), interferon γ (IFN-γ), and tumor necrosis factor α (TNF-α), and/or some proinflammatory stimuli such as lipopolysaccharides (LPS) triggers the activation of the transcription factors involved in the induction of iNOS gene expression (Donnelly and Barnes, 2002; Roy et al., 2004; Guo et al., 2016; Lee et al., 2017). On the other hand, guanidino-substituted analogs of L-arginine or methylarginines, such as asymmetric dimethylarginine (ADMA), inhibit the synthesis of NO by competing with L-arginine at the active site of NO synthases. Moreover, the arginase pathway can limit the arginine availability for NO synthesis by NO synthases (Rochette et al., 2013; Rath et al., 2014).

The iNOS induction generates large amounts of NO that is necessary to attack virus, bacteria, and tumoral cells between other functions. This is because, among other reactions, NO react with superoxide (O_2_^–^) and thus generate peroxynitrite (ONOO^–^) that, with other reactive oxygen species (ROS), damage several intracellular organelles and modify proteins and nucleic acids involved in the replication of tumoral cells, virus, and bacteria, supporting a key innate first defense of the organism (Mustafa et al., 2009).

**FIGURE 1:**
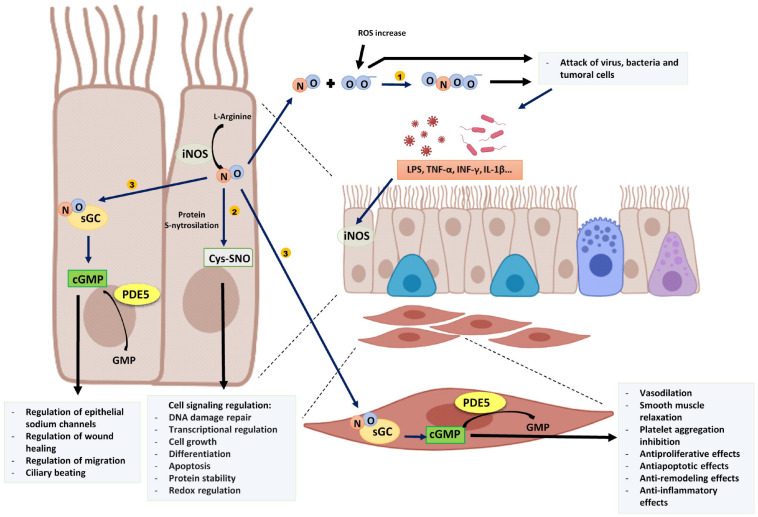
Proinflammatory stimuli and cytokines induce epithelial iNOS expression producing an increase of NO. (1) NO reacts with superoxide (O_2_^–^) and generates peroxynitrite (ONOO^–^) that, with other ROS damage tumoral cells and several intracellular organelles of pathogens. (2) NO is involved in several cell signaling pathways by protein S-nitrosylation. (3) NO binds to sGC of epithelial cells or other target cells such as muscle cells and produces cGMP. PDE5 degrades cGMP into GMP. The image has been created with Biorender.

In addition, NO is involved in many physiological processes mainly through cGMP-independent and cGMP-dependent pathways. The cGMP-independent actions of NO are frequently mediated by a post-translational modification of proteins by S-nitrosylation, which consists of the addition of a NO group to a cysteine thiol of a protein. This modification is involved in the regulation of protein conformation, interactions between proteins, and other post-translational modifications that activate or inhibit their function (Furuta, 2017). On the other hand, NO acts through the generation of cyclic guanosine-3′,5′-monophosphate (cGMP) after binding with soluble guanylate cyclase enzyme (Montfort et al., 2017) (Figure 1). The synthesized NO diffuses to target cells where it binds with picomolar affinity to the heme group of the active site of soluble guanylate cyclase increasing 100–200 times the catalytic activity of the enzyme and thus, the cGMP formation (Derbyshire and Marletta, 2012; Childers and Garcin, 2018), that can activate several kinases to implement cellular responses.

## Soluble Guanylate Cyclase–cGMP

The guanylate cyclases are enzymes that catalyze the guanosine 5′-triphosphate (GTP) conversion to cGMP, a second messenger that participates in several signaling processes (Dupont et al., 2014). There are two different types of guanylate cyclase enzymes. On the one hand, the particulate guanylate cyclase enzymes (pGC) are associated with plasmatic membrane and recognize different natriuretic peptides. On the other, the soluble guanylate cyclases (sGC) are localized in the cytoplasmatic region and are receptors of gaseous ligands, mainly nitric oxide (F. Rivero-Vilches et al., 2001).

The sGC enzymes are dimeric proteins formed by an α subunit (82 kDa) and a β subunit (70 kDa) (Rivero-Vilches et al., 2001). In human cells, there are two forms of the α subunit (α1, α2) and two forms of the β subunit (β1, β2). The active and best characterized forms are the α1/β1 and α2/β1 heterodimers (Haskó et al., 2006). Both heterodimers are present in the brain in similar proportions, however, the α1/β1 heterodimer is predominant in the rest of the tissues and is the most abundant in the lungs (Mergia et al., 2003). The group of Glynos et al. (2013) showed in lung sections that the α1 and β1 subunits are mainly present in bronchial and alveolar epithelial cells and in airway smooth muscle cells.

Both the α and β subunits polypeptides have four domains: a NO sensor N-terminal domain (H-NOX), a Per/Arnt/Sim domain (PAS domain), a coiled-coil domain, and a catalytic C-terminal domain (Derbyshire and Marletta, 2012). The catalytic domains at the C-terminus of both subunits are necessary for the binding and conversion of GTP to cGMP (Dupont et al., 2014).

**FIGURE 2:**
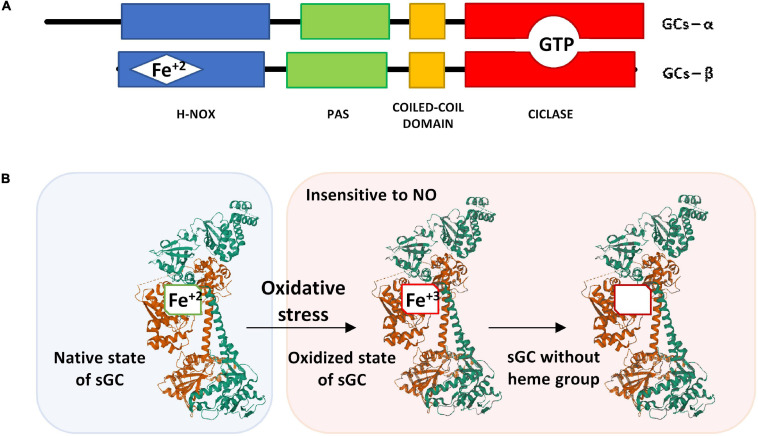
**(A)** Schematic representation of the α and β subunits of sGC. **(B)** Structure of the native state of sGC in its inactive form (without NO binding) and its oxidized form after oxidative stress. The α1 subunit is represented in green, the β1 subunit that contains the heme group is represented in brown. The image of the sGC has been created with Mol*, RCSB PDB: 6JT0 (Kang et al., 2019).

In the N-terminal domain of the β subunit, is the heme group attached to histidine 105. The heme group is formed by a protoporphyrin IX to which a ferrous ion is attached in its reduced redox form (Fe^+2^) (Figure 2A) (Iyer et al., 2003; Childers and Garcin, 2018). The NO binding to the reduced heme group (Fe^+2^) triggers a conformational change in the subunits structure, thus the enzyme catalytic effect is activated.

If the heme group is oxidized (Fe^+3^), the sGC enzyme is insensitive to NO (Figure 2B). Under these conditions, the heme group loses affinity for the enzyme and is released causing ubiquitination and proteolytic degradation of the protein (Dupont et al., 2014). In some lung diseases such as asthma and COPD in which oxidative stress is frequent, there is a loss of the heme group after its oxidation (Stasch et al., 2006) that causes a reduction of cGMP with consequences in the epithelial barrier that will be discussed in more detail below.

The increase of intracellular cGMP regulates several physiological processes, mainly by activating cGMP-dependent protein kinases (PKGs), phosphodiesterases (PDEs), and cGMP-dependent ion channels. The pathways involved in muscle relaxation, bronchi and blood vessels dilation, and inhibition of platelet aggregation are broadly described (Francis et al., 2010; Dupont et al., 2014). Furthermore, on the epithelial cells, cGMP is involved in signaling pathways of regulation of epithelial sodium channels related to airway and alveolar fluid clearance and differentiation, wound healing, migration, and ciliary beating, among others (Stout et al., 2007; Nie et al., 2009; Spitler et al., 2013; Liu et al., 2016).

## Phosphodiesterase 5 (PDE5)

The cGMP intracellular levels are regulated by the action of phosphodiesterases (PDEs) which rapidly degrade it to GMP. There are eleven characterized phosphodiesterases families (PDE1–PDE11) that specifically degrade cGMP, cyclic adenosine monophosphate (cAMP), or both (Francis et al., 2001). Furthermore, PDEs differ in their kinetic properties, their location at different tissues, and inside the cells and their sensitivity to certain drugs (Abusnina and Lugnier, 2017). The phosphodiesterases families PDE4, PDE7, and PDE8 are highly selective for cAMP, whereas the phosphodiesterases families PDE5, PDE6, and PDE9 are very selective for cGMP. The rest of them (PDE1, PDE2, PDE3, PDE10, and PDE11) degrade both (Francis et al., 2001).

Among PDEs families that catalyze cGMP, PDE5 regulates the cGMP balance in multiple tissues and is abundantly expressed in the lungs (Corbin et al., 2005; Shafiee-Nick et al., 2017) in which it plays an important role in the cGMP metabolism of epithelial cells (Fuhrmann et al., 1999). PDE5 inhibitors have been used to treat several diseases, for instance, the drug called sildenafil is approved for the treatment of erectile dysfunction or pulmonary arterial hypertension because it induces smooth muscle relaxation. Additionally, the use of PDE5 inhibitors is being investigated to treat other pathologies (Dupont et al., 2014) such as in CF, in which PDE5 inhibitors might correct abnormalities on transepithelial ion transport (Noel et al., 2012).

## Role of Nitric Oxide on the Regulation of Immune Responses

Such as mentioned above, the iNOS gene promoter is very complex and differs between different species and cell types. iNOS expression is activated by several cytokines or stimuli after recognition by epithelial receptors, such as Toll-like Receptor 4 (TLR4) in the case of LPS (Jia et al., 2016), INFγ receptor, TNF receptor or IL-1 receptor. In epithelial cells, IL-1β and TNF-α stimulation induce the activation and translocation into the nucleus of nuclear transcription factor κB (NF-κB). However, INF-γ stimulation activates STAT-1 and IRF-1 (Lee et al., 2017). The synergic effect between IL-1β, TNF-α, and INF-γ is due in part to different mechanisms. Apart from the NF-κB activation, IL-1β and TNF-α are involved in the BH4 synthesis, an essential cofactor for iNOS activity. On the other hand, INF-γ interacts with IL-1β to enhance the degradation of the inhibitor of nuclear factor κB (IκB). Finally, they activate different iNOS promoters enhancing iNOS expression (Kwon et al., 2001). AP-1 is another important transcription factor for iNOS expression in airway epithelial cells. Stimulation with LPS and INF-γ activates mitogen-activated protein kinase (MAPK) pathways enhancing the binding of AP-1 protein to specific promoter sequences. However, LPS alone cannot activate iNOS expression, and although INF-γ alone can activate its transcription, the addition of other cytokines and coactivators can potentiate iNOS expression and activation (Guo and Erzurum, 1998; Kristof et al., 2001). The coactivator p300 might be essential to the iNOS activation since, after stimulation with TNF-α, IL-1β, and IFN-γ cytokines, allows the formation of a long-range DNA looping between AP-1 and TATA box of iNOS promoter stabilizing the transcription complex and activating gene transcription (Guo et al., 2016). Finally, the INF-JAK-STAT pathway plays also an important role in the induction of iNOS expression since inhibition of JAK signaling inhibits iNOS cytokine-induced expression in airway epithelial cells after TNF-α, IL-1β, and IFN-γ stimulation (Ganster et al., 2001).

NO is a key molecule in the primary host defense. As mentioned above, NO after reaction with other ROS, has cytotoxic effects essential to attack virus and bacteria and to prevent pathogen infection. Additionally, NO is involved in the S-nitrosylation of cysteine residues of vital pathogen enzymes. Among its antimicrobial effects, NO has shown antiviral effects against DNA and RNA viruses, including SARS-CoV-2, by partially inhibiting virus replication (Rolim et al., 2019; Akaberi et al., 2020; Pieretti et al., 2021). However, it has been shown that viral activity can also compromise host NO production (Ritz et al., 2021). Finally, NO is an important modulator of epithelial ciliary beating, important for the clearance of pathogens, through the activation of the sGC-GMPc-PKG pathway (Li et al., 2000).

NO is also involved in the regulation of various signaling pathways related to transcription factor activation and gene expression and in posttranslational regulation of the activity of various inflammatory mediators. Among the mediators regulated by NO, NF-κB is a key mediator in the airway epithelial inflammatory response. NF-κB is both increased or decreased after NO exposure depending on the NO concentration and the time of exposure. Elevated NO levels after iNOS induction increase NF-κB activation through cGMP-dependent and independent pathways. However, NO may inhibit NF-κB activation through a feedback mechanism to avoid prolonged NF-κB activation and inflammation. Furthermore, the effects of its activation are complex (Bove and van der Vliet, 2006). In airway epithelial cells, NO increases IL-8 expression via cGMP independent pathways but ERK and protein kinase C dependent pathways involving AP-1 and NF-κB transcription factor activation. These results highlight the importance of NO activation of IL-8 in the initiation of an inflammatory response in the airway epithelium since IL-8 is up-regulated in several chronic pulmonary inflammatory diseases (Sparkman and Boggaram, 2004). After IL-1β stimulation of airway epithelial cells, NF-κB, AP-1, and MAPK activation leads to increased metalloproteinase 9 (MMP-9) expression (Lin et al., 2009). Additionally, in epithelial cells, NF-κB is also involved in the activation of cyclooxygenase-2 (COX-2) and consequently prostaglandin E2 (PGE2), two significant factors in the development of inflammatory diseases such as asthma. In this work, inhibition of NF-κB also downregulated the expression of several cytokines such as IL-4, IL-6, and eotaxin involved in asthmatic pathology (Lee et al., 2018).

In addition, NO is also involved in leukocyte chemotaxis and infiltration. High NO concentrations after iNOS stimulation inhibit protein adhesion expression on endothelial cells due to S-nitrosylation of p50 and p65 in NF-κB and IKKβ (Aguilar et al., 2020). Furthermore, NO also acts via cGMP dependent pathway. Activation of sGC plays an important anti-inflammatory role by inhibiting leukocyte recruitment after inhibition of P-selectin expression on endothelial cells and platelets preventing leukocyte rolling and adhesion (Ahluwalia et al., 2004; Thomazzi et al., 2005).

NO also regulates the adaptive immune response and links the innate and the adaptive immunity (Bingisser and Holt, 2001). The results obtained about the role of NO in T cell differentiation are controversial. The most important cytokines that induce T-helper 1 (Th1) or T-helper 2 (Th2) differentiation are IL-12 for Th1 and IL-4 for Th2. Low concentrations of NO induce the production of IL-12Rβ2 in human T cells favoring Th1 differentiation and proliferation via cGMP-dependent pathways. However, high concentrations of NO inhibit Th1 responses by decreasing the IL-12 production of macrophages. Therefore, NO might regulate the balance between Th1 and Th2 depending on its concentration by increasing Th1 apoptosis at high concentrations and inhibiting it at low concentrations (Ibiza and Serrador, 2008; Lee et al., 2017). In contrast to this data, the addition of NO to bronchial epithelial cells showed a reduction in both Th1 and Th2 proliferation. NO induced cGMP mediated STAT5 dephosphorylation that interferes with the IL-2R signaling cascade involved in T cell proliferation (Eriksson et al., 2005). However, NO is also involved in T cell differentiation at the transcriptional level and high levels of NO might activate Th2 transcription factor STAT-6 and GATA-3 upregulating IL-4 mediated Th2 cell differentiation (Ibiza and Serrador, 2008). Although the play of NO in T cell differentiation is not fully elucidated, NO participates in Th1/Th2 balance playing an important role in several diseases such as asthma in which there is a Th2 chronic inflammation. In asthma, Th2 cells produce several cytokines such as IL-5 involved in the recruitment of eosinophils which in turn produce chronically inflammatory mediators leading to the loss of epithelial integrity (Barnes, 2008), a process that will be described in more detail below.

## Role of Nitric Oxide System in Bronchial Epithelium and Related Diseases

Although in healthy conditions NO has beneficial effects by regulating various biological processes related to airway function and maintains lung homeostasis, dysregulation of the NO concentration has pathologic effects and contributes to various pulmonary diseases (Barnes et al., 2010; Garren et al., 2021).

NO participates in several signaling pathways and suboptimal levels of NO in the lungs are pathological because these pathways become altered. However, an excess of NO and the consequences of its combination with ROS, such as the formation of peroxynitrite, have also a pathological impact. The most specific reaction of peroxynitrite is a post-translational modification of tyrosine residues of proteins, generating 3-nitrotyrosine (3-NT) or tyrosine nitrated proteins. Although protein tyrosine nitration occurs in physiological conditions, dysregulation of this process due to inflammatory responses and oxidative stress is related to several diseases, including lung diseases (Yeo et al., 2008; Ahsan, 2013). Protein tyrosine nitration causes changes in the protein structures, altering their conformation and function. For example, after tyrosine nitration of PKG, its enzymatic activity is decreased and the binding to cGMP is changed. In addition, protein nitration can interfere in tyrosine phosphorylation and dephosphorylation, regulating cellular signal transduction processes mediated through kinases and phosphatases. Finally, this post-translational modification may generate unmasking of epitopes triggering an immune response. Consequently, the accumulation of nitrated proteins in apoptotic and inflamed tissues due to oxidative stress may induce an autoimmune response aggravating the chronic inflammatory response (Thomson et al., 2007; Abello et al., 2009; Sabadashka et al., 2021).

### Role of Nitric Oxide System in Bronchial Epithelium of Asthma and COPD Patients

Asthma and COPD are chronic respiratory diseases characterized by chronic inflammation in the lungs and airway obstruction, which is generally reversible in asthma but irreversible and progressive in COPD. Although the nature of the inflammation is not the same between both diseases, they share characteristics, since many of the cytokines and chemokines that are secreted in COPD and asthma are regulated by NF-κB, which is found activated in airway epithelial cells and macrophages in both diseases. Moreover, chronic activation of these mediators also contributes to structural changes named airway remodeling that is characteristic of these pathologies (Barnes, 2008; Gao et al., 2015). This airway remodeling is responsible for irreversible airway narrowing and airflow limitation and is caused by repeated cycles of injury and repair. In asthmatic patients, this airway remodeling is mainly caused by an increase of airway smooth muscle mass, but also is characterized by epithelial cell hyperplasia, goblet cell metaplasia, angiogenesis, and basement membrane thickening caused by deposition of extracellular matrix proteins (Grigoraş et al., 2016). Airway inflammation also contributes to airway obstruction by promoting mucus overproduction. In asthma, the expression of MUC5AC is upregulated together with stimulated mucin secretion (Evans et al., 2009). Finally, inflammation is also related to bronchial hyperresponsiveness, an exaggerated reduction in airway caliber after stimuli such as allergens or pollutants, among others (McCracken et al., 2017). In COPD patients, emphysema, destruction and loss of the alveoli, is related to small-airway obstruction and is one of the principal characteristics of the disease (McDonough et al., 2011). The small airway narrowing is caused by peribronchial fibrosis, thickening of the basement membrane, collagen deposition, epithelial cell hyperplasia, squamous and goblet cell metaplasia, and angiogenesis (Hirota and Martin, 2013). Finally, it is observed ciliary dysfunction and mucus hypersecretion that also contributes to airway obstruction (Barnes, 2017).

**FIGURE 3:**
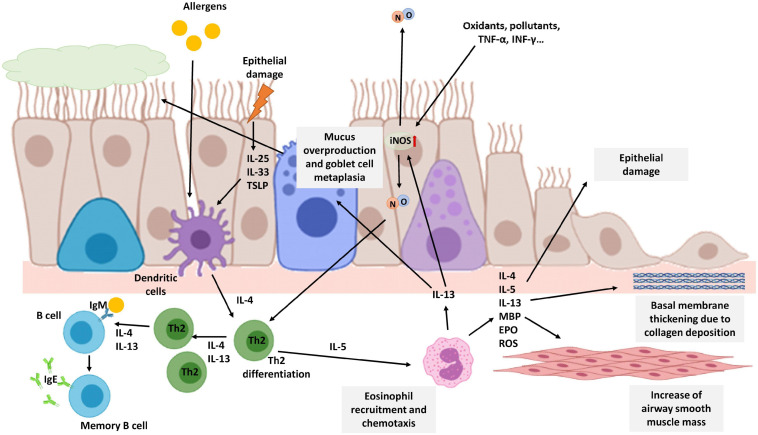
Schematic view of T2 eosinophilic airway inflammation in the pathophysiology of asthma. Allergens or epithelial damage activates dendritic cells that secrete cytokines, such as IL-4, leading to Th2 differentiation. Th2 lymphocytes secrete IL-4 and IL-13 amplifying Th2 proliferation and promoting the generation of IgE by B lymphocytes. Th2 cells also secrete IL-5, the most important cytokine for eosinophil recruitment. IL-13 secreted mainly by eosinophils activates iNOS expression increasing NO levels in the airways and consequently F_E_NO. NO, in turn, is also involved in Th2 differentiation. Moreover, iNOS expression on epithelial cells could be also enhanced by oxidants, pollutants, or proinflammatory stimuli such as TNF-α or INF-γ. Chronic eosinophil inflammation is involved in tissue airway remodeling and bronchial obstruction caused by an increase of airway smooth muscle mass, epithelial cell hyperplasia, goblet cell metaplasia, mucus overproduction, and basement membrane thickening caused by deposition of extracellular matrix proteins. The image has been created with Biorender.

Asthma has a very heterogeneous clinical spectrum, but it is characterized as a chronic inflammatory disease of the airways in which various cells and inflammation mediators participate. Generally, asthma is considered allergic, but this endotype is only prevalent in 40–60% of adult patients (Pakkasela et al., 2020). Patients with allergic asthma are atopic and have an allergic inflammation pattern. This type of asthma is called Type 2 (T2) asthma because it is orchestrated by Th2 lymphocytes that secrete a series of interleukins such as IL-4, −5, −9, and −13, which cause activation and recruitment of eosinophils, as well as the generation of IgE by B lymphocytes (Figure 3) (Barnes, 2017). In asthma patients, especially T2 asthma patients with eosinophilic airway inflammation, NO levels in exhaled air are higher compared to levels in healthy patients. Furthermore, higher production of NO is correlated with higher airway obstruction (Comhair et al., 2015; Xu et al., 2017; Asosingh et al., 2020).

This increase in the fraction of exhaled NO (F_E_NO) in patients with asthma is mainly caused by an increase in the expression and activity of the iNOS enzyme due to pro-inflammatory stimuli: cytokines, oxidants, and other inflammatory mediators. In the activation of iNOS expression, eosinophils are essential since they secrete IL-13. This cytokine increases iNOS expression in epithelial cells and consequently, NO levels and F_E_NO. However, in F_E_NO measurements is difficult to differentiate between constitutive NO and the NO produced after an allergic inflammation. In asthmatic patients not treated with steroids, this increased expression has been observed mainly in bronchial epithelial cells and in macrophages of the alveolar region (Roos et al., 2014; Sato et al., 2019). Furthermore, a correlation between F_E_NO and bronchial wall thickening has been observed in asthma patients (Nishimoto et al., 2017).

On the other hand, COPD is a disease caused mainly by tobacco consumption, a source of exogenous NO. Tobacco smoke contains many harmful substances that cause an inflammatory response and excessive oxidative stress in the lungs (Milara and Cortijo, 2012; Miravitlles et al., 2017). This large amount of ROS in the lungs of COPD patients not only amplifies the inflammatory response, but also induces the remodeling of the airways and cell death of structural cells in the lung that causes emphysema (Brusselle et al., 2011).

**FIGURE 4:**
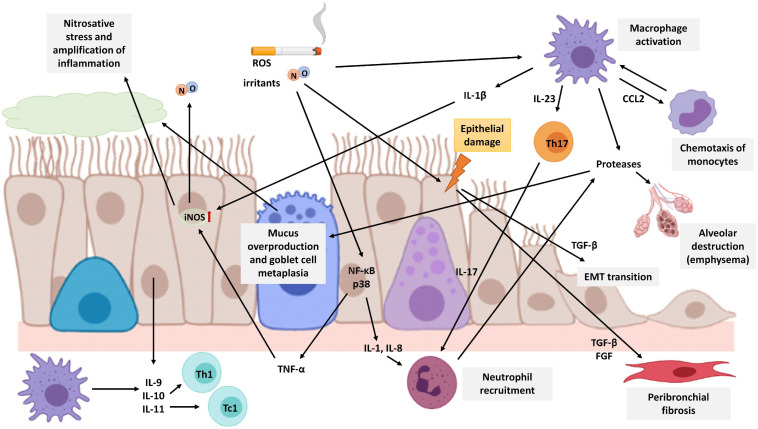
Schematic representation of lung neutrophilic inflammation characteristic of COPD. Cigarette smoke is a source of exogenous NO, irritants, and ROS that activates macrophages and epithelial cells of the airways to release cytokines that attract inflammatory cells to the lungs. Macrophages secrete CCL2 to attract monocytes which differentiate into macrophages in the lungs. Epithelial cells secrete IL-1 and IL-8 to attract neutrophils, and both macrophages and epithelial cells secrete IL-9, IL-10, and IL-11 to attract Th1 cells and Tc1 cells. In addition, macrophages also release IL-23 triggering Th17 cell activation which in turn promotes neutrophilic inflammation by producing IL-17. Neutrophils, macrophages, and epithelial cells release proteases, such as MMP-9, which cause alveolar destruction, emphysema, mucus overproduction, and goblet cell metaplasia. Cigarette smoke causes epithelial damage that triggers the epithelial cell secretion of TGF-β, among other growth factors, which stimulates fibroblast proliferation and EMT, resulting in airway remodeling and fibrosis around the small airways. The expression of the iNOS enzyme is increased in epithelial cells by TNF-α and IL-1β produced by epithelial cells and macrophages, respectively. Increased NO levels are associated with epithelial-cell-derived nitrosative stress, which causes oxidation and tyrosine nitration of several lung proteins generating an amplification of the inflammatory response. The image has been created with Biorender.

COPD patients have exaggerated chronic inflammation with increased numbers of neutrophils and macrophages in the lumen of the airways. In addition, there is also an increase in macrophages and T and B lymphocytes in the wall of the airways and in the parenchyma (Figure 4) (Brusselle et al., 2011; Barnes, 2017). In COPD, epithelial cells are an important source of inflammatory mediators and proteases and are an important source of transforming growth factor β (TGF-β), a growth factor linked to airflow limitation in small conducting airways and in fibrosis, initiating a perpetuating peribronchial fibrosis remodeling that contributes to small airway obstruction (Milara et al., 2013). In vitro stimulation of human bronchial epithelial cells with cigarette smoke extract showed an increase in activation of ROS, a major release of TGF-β1, and increased phosphorylation of ERK1/2 and Smad3. All of them are related to epithelial to mesenchymal transition (EMT) and contribute to the thickening of the wall of the small airways (Milara et al., 2013).

In addition, it has been observed that F_E_NO levels in COPD patients are higher than the levels of healthy non-smokers, however, these levels are not as high as those observed in asthmatic patients before their treatment (Ansarin et al., 2001). The expression of the iNOS enzyme is increased in the peripheral lung tissues of COPD patients and is associated with epithelial-cell-derived nitrosative stress, which causes oxidation and tyrosine nitration of several lung proteins generating an amplification of the inflammatory response. In addition, iNOS expression is related to the degree of airflow limitation in the airways (Ghosh et al., 2006; Jiang et al., 2015; Ricciardolo et al., 2015; Bartesaghi and Radi, 2018). The group of Fysikopoulos et al. (2020) established a mouse model of emphysema by treatment with elastase, and after pharmacological inhibition of iNOS, it demonstrated a partial regeneration of the parenchyma, so there is a relationship between increased expression of the enzyme and the appearance of emphysema, although it would not be the only cause.

Moreover, although there is an increased NO production by the epithelial cells, an increase in the activation of the lung sGC is not observed in COPD and asthma, and therefore there is not an increase in cGMP (Dupont et al., 2014). The NO-sGC-cGMP signaling pathway can be affected for various reasons. Firstly, there is a reduction in the amount of intracellular availability of NO due to its reaction with O_2_^–^ for the generation of peroxynitrite. Secondly, due to oxidative stress in the lung, the redox state of sGC is altered (Fe^+2^ → Fe^+3^), which makes it inactive to the binding of NO (Haskó et al., 2006). In addition, decreased expression of the sGC enzyme has been observed in alveolar and bronchial epithelial cells and in airway smooth muscle cells in COPD patients and smokers (Glynos et al., 2013; Weissmann et al., 2014), as well as in asthmatic patients and animal models of asthma (Papapetropoulos et al., 2006; London et al., 2018). The underlying mechanism is not well understood but recently, it has been shown that the expression of the α1 subunit of sGC is downregulated by TGF-β in pulmonary artery smooth muscle cells via MEK and ERK signaling (Du and Roberts, 2019) and IL-1β in perinatal lung fibroblasts via TAK1 and NF-κB signaling (Zhong et al., 2020) and both inflammatory mediators are increased in COPD and asthma disease so they might be also involved on the reduction of α1 subunit of sGC in the epithelial cells of these patients.

A lower expression of sGC as well as a lower activity due to their oxidation, generate less cGMP. This fact causes less activation of PKG and consequently an increase in TGF-β signaling related to an increase in the tone of the airways and fibrosis (Verrecchia and Mauviel, 2007). This is because TGF-β acts through two signaling pathways: the classical pathway, also called canonical, dependent on SMAD, and the non-canonical pathway independent from SMAD (Hu et al., 2017). The production of cGMP interferes with TGF-β signaling mainly through the activation of PKG, which inhibits the independent SMAD pathway. This inhibition of the non-canonical pathway is critical in COPD and asthma in which TGF-β activates epithelial cells that change their phenotype to mesenchymal cells (Willis and Borok, 2007; Hackett et al., 2009; Sohal et al., 2014). As previously mentioned, this process called EMT contributes to airway remodeling since epithelial cells lose cell-cell adhesion and cell polarity. Epithelial cells show decreased epithelial markers, such as E-cadherin and occludin, in the EMT process. Meanwhile, they show an increased expression of mesenchymal proteins, such as vimentin and alpha-smooth muscle actin (α-SMA), and increased synthesis and secretion of proteins of the extracellular matrix such as collagen I (Hackett et al., 2009; Johnson et al., 2011; Milara et al., 2013).

### Role of Nitric Oxide System in Bronchial Epithelium of CF Patients

CF is a chronic inflammatory disease caused by a genetic defect of the CF transmembrane conductance regulator (CFTR) gene that results in abnormal chloride-ion transport by epithelial cells (Rout-Pitt et al., 2018). There are more than 1,400 mutations that can produce CF but the absence of a phenylalanine at position 508 of the CFTR polypeptide is the most frequent (Boucher, 2007). Mutations on the CFTR gene have also negative effects on other ion transporters. One of the most remarkable is the loss of inhibition of the amiloride-sensitive epithelial sodium channel (ENaC) in lung epithelial cells of CF patients and in consequence an organellar hyper-acidification in these cells responsible for protein glycosylation among other functions (Poschet et al., 2002). In addition, this failure on the inhibition of the ENaC causes dehydration and reduction of the airway surface liquid (ASL) affecting the mucociliary clearance function of the epithelial cells (Mroz and Harvey, 2019) and thus, producing mucus accumulation that causes airway obstruction.

Disability of the mucociliary clearance is related to continual bacterial infection (especially *P. aeruginosa*) and neutrophilic inflammation (De Rose et al., 2018; Cabrini et al., 2020). In this neutrophilic inflammation, bronchial epithelial cells are crucial due to their secretion of cytokines, being IL-8 the most important, that recruit neutrophils to bronchi and bronchioles. However, neutrophils have also mutated the CFTR gene and are defective. Consequently, neutrophils cannot eliminate the bacterial infection, worsening the disability of the mucociliary clearance and chronically releasing proteases and ROS that contributes to airway tissue damage and remodeling (Cabrini et al., 2020).

Young infants with CF show a reduced F_E_NO, and this reduction is higher in infants without CFTR function (Korten et al., 2018). This is related to dysfunction in the bronchial epithelium of CF patients that express lower levels of iNOS compared with healthy patients (Meng et al., 1998). This lack of NO in CF patients has several consequences in the patients.

Firstly, NO has antimicrobial properties and reduces the sequestration of polymorphonuclear leukocytes (Sato et al., 1999), so these low levels of NO could be related to the major neutrophil infiltration of the disease. CF bronchial epithelial cells co-cultured with neutrophils (Meng et al., 2000) or stimulated with cytokines (Meng et al., 1998) showed no increase in iNOS expression in contrast with normal bronchial epithelial cells, suggesting that this lack of NO plays an important role in bacterial colonization and neutrophil infiltration.

**FIGURE 5:**
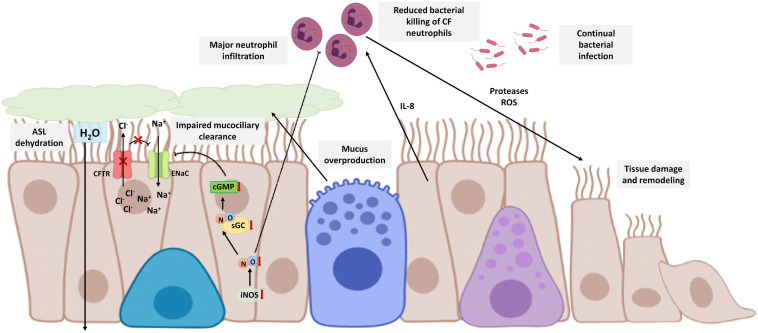
Schematic view of CF bronchial epithelial cells and neutrophilic inflammation. CFTR defective protein results in mucus overproduction, a decrease of chloride-ion transport, and an increase of sodium transport through the no inhibition of ENaC. Therefore, there is dehydration and reduction of ASL that affects mucociliary clearance. CF epithelial cells express lower levels of iNOS in comparison with healthy epithelial cells and consequently suboptimal cGMP levels that contribute with the no inhibition of ENaC. On the other hand, the disability of the mucociliary clearance is related to continual bacterial infection. Bronchial epithelial cells secrete cytokines, such as IL-8, that recruit neutrophils to bronchi and bronchioles. Neutrophils are CFTR defective with reduced bacterial killing, worsening the disability of the mucociliary clearance, and chronically releasing proteases and ROS that contributes to airway tissue damage and remodeling. NO reduces the sequestration of polymorphonuclear leukocytes so that lower levels of NO contribute to the major neutrophil infiltration. The image has been created with Biorender.

On the other hand, this reduction of the NO levels involves a reduction of sGC activity and in consequence a decrease of cGMP levels. In healthy conditions, cGMP participates in the inhibition of the ENaC. However, in CF patients, this suboptimal cGMP formation contributes to maintaining the chronic activation of ENaC characteristic of the disease (Figure 5). As previously mentioned, this sustained ENaC activation is related to hyper-acidification in CF cells, defective protein glycosylation, bacterial adherence, proinflammatory responses, and ASL dehydration related to an impairment of mucus secretion and mucociliary clearance (Poschet et al., 2007; Reihill et al., 2016). In addition, lower cGMP also aggravates the disability of mucociliary clearance by disruption of the NO-sGC-cGMP-PKG pathway (Jiao et al., 2011).

### Role of Nitric Oxide in Bronchial Epithelium of Cancer Patients

According to the World Health Organization (WHO) lung cancer is the first cause of cancer death worldwide and, such as in COPD, tobacco smoking (source of NO and ROS) is the main risk factor for lung cancer development (Bade and Dela Cruz, 2020). In patients with lung cancer, a loss of epithelial integrity due to changes in intercellular adhesions and cell polarity have been observed, which leads to changes in expression of genes related to differentiation, proliferation, and apoptosis and in consequence development of dysplasia and malignant transformation (Bonastre et al., 2016; Zhou et al., 2018). In addition, cell adhesions play an important role in cancer metastasis, a process in which epithelial cells lose their cell-cell contacts and their morphology and migrate to a distant site forming a new tumor (Yilmaz and Christofori, 2010; Rusu and Georgiou, 2020).

NO has shown cancerogenic or anti-cancerogenic effects depending on the concentration and duration of its presence, the microenvironment, the localization, and the cellular targets (Korde Choudhari et al., 2013; Alimoradi et al., 2019). Patients with lung cancer show higher levels of F_E_NO than healthy controls (Liu et al., 2018), and in line with this, Masri et al. (2005) observed an elevated NO, nitrite, and nitrotyrosine in cancer patients. The nitration occurs mainly in proteins related to oxidant defense, energy production, structure, and apoptosis and may contribute to several cancer-related pathways (Masri et al., 2005). Furthermore, it has been demonstrated that high levels of serum nitrite/nitrate are associated with advanced-stage lung cancer and a lower survival rate of patients and this suggests that NO microenvironment and signaling is implicated in the pathophysiology of cancer, particularly in aggressive tumor phenotypes and metastasis (Colakogullari et al., 2006).

In physiological conditions, after DNA damage, NO activates p53 inducing apoptosis of cells (Meßmer et al., 1994). However, an excess of NO inactivates p53 function in several types of cancer. Firstly, an excess of NO is related to GC to AT mutations in the p53 gene in non-small cell lung cancer (NSCLC) that leads to p53 loss of function (Fujimoto et al., 1998; Marrogi et al., 2000). In addition, after exposing malignant glioma cells to peroxynitrite and breast cancer cells to NO donors, a posttranslational modification by tyrosine nitration of p53 has been demonstrated (Chazotte-Aubert et al., 2000; Cobbs et al., 2003). Moreover, NO production in tumors by iNOS could promote cancer progression by providing a selective growth advantage to tumor cells with loss of p53 repressor function (Ambs et al., 1998). All these observations may be transferable to lung cancer since more than 90% of lung tumors are p53 defective (Masri et al., 2005). Higher concentrations of NO in the lung are also associated with a downregulation of caspase-3 activity (Chen et al., 2008) and S-nitrosylation and stabilization of BCl-2 protein (Azad et al., 2006), both of them contributing to the suppression of apoptosis pathways.

Furthermore, NO is also involved in the loss of epithelial cell adhesions and EMT that has been mentioned above, a key process related to cancer cell migration, invasion, and metastasis. Lung cancer cells increase EMT and thus cell migration after NO prolonged stimulation, by increasing vimentin and snail expression and decreasing E-cadherin levels (Chanvorachote et al., 2014; Yongsanguanchai et al., 2015). In addition, NO also enhances epithelial cell migration by caveolin-1 upregulation (Sanuphan et al., 2013; Chanvorachote et al., 2014).

**FIGURE 6:**
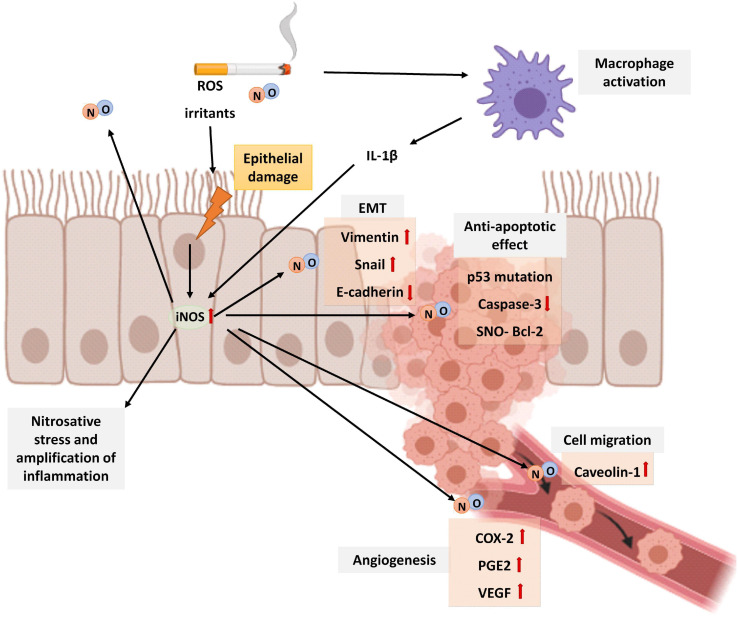
Schematic representation of the role of NO in lung cancer. Cigarette smoke is a source of exogenous NO, irritants, and ROS that activates macrophages and epithelial cells of the airways to release cytokines that attract inflammatory cells to the lungs. In epithelial cells, the expression of the iNOS increases by proinflammatory stimuli such as IL-1β produced by macrophages. Patients with lung cancer show higher levels of F_E_NO than healthy controls. An elevated NO generates nitrosative stress and amplification of inflammation. Although in physiological conditions, after DNA damage, NO activates p53 inducing apoptosis of cells, an excess of NO inactivates p53 function. In addition, higher concentrations of NO in the lung also downregulates caspase–3 activity and S-nitrosylation and stabilization of BCl-2 protein, all of them contributing to inhibition of apoptosis. Prolonged NO stimulation is additionally related to EMT by increasing vimentin and snail expression and decreasing E-cadherin levels. NO also enhances epithelial cell migration by caveolin-1 upregulation and angiogenesis by COX-2, PGE2, and VEGF upregulation. The image has been created with Biorender.

Finally, in NSCLC, it has been shown a correlation between iNOS levels and activation of COX-2, PGE2, and vascular endothelial growth factor (VEGF), all of them related to induction of angiogenesis and thus with tumor progression (Marrogi et al., 2000; Korde Choudhari et al., 2013) (Figure 6).

## Pharmacological Modulation of iNOS-NO-sGC- cGMP Axis

**FIGURE 7:**
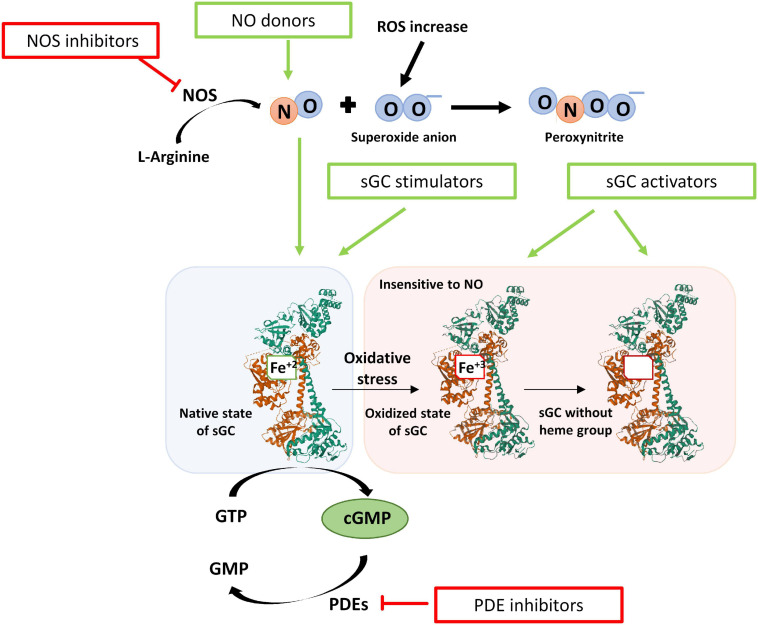
Scheme of the redox state of the sGC enzyme and the modulatory drugs that act on the NO- sGC-cGMP pathway. After oxidative stress, the heme group is oxidized (Fe^+3^), and the sGC enzyme is insensitive to NO. In addition, the oxidized heme group loses affinity for the enzyme and is released. The drugs that can modulate this axis are NO donors, iNOS inhibitors, PDE5 inhibitors, and sGC modulators. sGC modulators increase the activity of sGC and thus the formation of cGMP independently of NO and are classified as stimulators or activators of sGC. Stimulators of sGC act when the heme group is reduced (Fe^+2^). Activators of sGC can activate the enzyme even when the heme group is oxidized (Fe^+3^) or lost.

Modulation of the NO-sGC-cGMP axis offers a therapeutic arsenal for the treatment of the mentioned diseases (Figure 7). Among the modulating drugs of this pathway, there are NO donors, iNOS inhibitors, PDE5 inhibitors, and sGC stimulators and activators (Dupont et al., 2014).

### NO Donors

NO donor drugs, such as organic nitrates, are limited as a treatment for COPD and asthma. As previously explained, NO reacts also with other biological molecules, for example generating peroxynitrite. Indeed, NO levels are already elevated in these pathologies, but the activity of the sGC enzyme is not increased (Haskó et al., 2006).

However, NO donors may be a potential treatment for CF patients since they increase the chloride-ion efflux and decrease the expression of ENaC subunits from bronchial CFTR defective epithelial cells. Both actions may lead to the restoration of hydration of the airways of these patients (Oliynyk et al., 2013). In addition, NO donors may also improve ciliary beating function so, together with the enhanced airways hydration, may benefit the mucociliary clearance (Li et al., 2000; Oliynyk et al., 2013). Among NO donors, S-nitrosoglutathione (GSNO) is an endogenous S-nitrosothiol, source of bioavailable NO. GSNO apart from increase chloride-ion efflux via cGMP-dependent and independent pathways (Chen et al., 2006), also increases the expression, maturation, and cell-surface expression stabilization and function of CFTR in human bronchial airway epithelial cells (Zaman et al., 2014). Nevertheless, it is an unstable compound since the enzyme GSNO reductase (GSNOR) reduces it to an unstable intermediate, regulating the concentration of GSNO and thus the bioavailability of NO. For that reason, other compounds, that inhibit the activity of GSNOR and thus increases levels of GSNO have being investigated for CF treatment (Quon and Rowe, 2016). In fact, inside this group, cavosonstat (N91115) is in phase II study (NCT02724527).

Due to the dichotomous role of NO in cancer, NO donors could give pharmaceutical options for cancer therapy as chemoadjuvants if the appropriate concentration reaches the tumor (Alimoradi et al., 2019). In A549 epithelial cells, NO donors enhanced the cytotoxicity of pemetrexed via cGMP-dependent pathways (Nagai et al., 2012). Nitroglycerin is in two phase II studies for the treatment of NSCLC in combination with radiotherapy and/or chemotherapy (NCT01210378, NCT00886405). In addition, due to the necessity to control NO delivery, NO-releasing vehicles are being investigated (Alimoradi et al., 2019). Nanoparticles loaded with nitric oxide and cisplatin have been developed for the treatment of NSCLC and shows higher cytotoxic effect in cancer cells than nanoparticles only loaded with cisplatin (Munaweera et al., 2015).

### iNOS Inhibitors

iNOS inhibitor drugs are able to reduce the NO excessively produced by iNOS, which reacts quickly to produce peroxynitrite, but would also reduce the beneficial effect of the activation of sGC. There are disparate results seen for the treatment of emphysema and asthma patients with iNOS inhibitors. In a mouse model with emphysema, after the inhibition of iNOS was observed a significant regeneration of the lung (Fysikopoulos et al., 2020), but these results contrast with those obtained by the group of Boyer et al. (2011) in which inhibition of iNOS activity reduced protein nitration and protein oxidation without effect on inflammation, proliferation, and development of emphysema. These discrepant results are probably due to the degree of damage provoked by the elastase treatment applied to induce emphysema and the time of treatment with the iNOS inhibitor. Boyer et al. (2011) used a more aggressive dose of elastase that generated more alveoli destruction, and they also applied the iNOS inhibitor for a shorter duration than the group of Fysikopoulos et al. (2020). These results suggest that the iNOS inhibitors could be a therapeutical option for early lung emphysema but not for more severe emphysema.

iNOS inhibitors reduce F_E_NO in patients with asthma, but that fact did not improve hyper-reactivity or the number of inflammatory cells (Singh et al., 2007). However, in animal models of asthma with acute but not chronic allergen exposure iNOS inhibition was related to a reduction in hyperresponsiveness (Ibba et al., 2016).

In mouse lung tumors has been shown that epithelial cells at the periphery of lung tumors had a significant expression of iNOS suggesting an important role of NO in tumor growth. Moreover, the genetic ablation of the iNOS gene decreases 80% the lung tumor development in mice (Kisley et al., 2002). In line with these results, in a mouse model of NSCLC with mutations on the p53 and KRAS genes was shown that administration of the NOS inhibitor L-NAME inhibited lung tumor growth, reduced tumor burden, and improved survival (Pershing et al., 2016). The iNOS inhibitor ASP9853 in combination with docetaxel showed a major growth inhibition than docetaxel alone against NSCLC. However, due to toxicity and lack of significant efficacy, the study of the combined drug effect was stopped in clinical phase I (Luke et al., 2016). Currently, the iNOS inhibitor L-NMMA in combination with pembrolizumab, is in clinical phase I study for the treatment of NSCLC and small cell lung cancer (SCLC), among other cancer types (NCT03236935).

### PDE5 Inhibitors

PDE5 inhibitors such as sildenafil, vardenafil, or tadalafil are used in diseases such as erectile dysfunction, pulmonary hypertension, and cardiovascular diseases for their smooth muscle relaxation effect (Sandner, 2018). In COPD or asthma, these types of drugs have shown an anti-inflammatory effect (Mokry, 2017; Ren et al., 2020). Moreover, apart from reducing airway inflammation, sildenafil attenuates the mucus overproduction characteristic of both diseases through the restoration of cGMP levels (Wang et al., 2009). In an animal model of COPD, sildenafil showed a reduction in lung damage. After exposure to tobacco smoke and bacterial inhalation, these animals showed an increase in both proliferation and apoptosis pathways in epithelial cells of bronchioles, suggesting that the pulmonary damage is related to the abnormal repair of the airway epithelium. Treatment with sildenafil significantly reduces the apoptosis in the bronchiolar epithelium reducing the pulmonary damage (Ren et al., 2020). These results are in line with others that suggest that inhibition of PDE5 can alleviate lung dysfunction and tobacco smoke-induced emphysema with the restoration of the NO-sGC-cGMP-PKG pathway and reduction of ROS (Milara et al., 2010; Seimetz et al., 2015). However, its efficacy is limited in COPD and asthma since the sGC activation is decreased and, therefore, cGMP levels are also decreased. In these cases, although the degradation of cGMP is inhibited, sufficient levels are not reached for the treatment of these pathologies (Evgenov et al., 2011; Sandner, 2018).

In mutated F508del CF mice, inhaled exposure of the PDE5 inhibitors sildenafil, vardenafil, and tadalafil, leads to restoration of chloride transport across the respiratory epithelium (Lubamba et al., 2011). Sildenafil acts in two ways in human bronchial epithelial cells: via cGMP-dependent and cGMP-independent pathways. Through the cGMP-dependent pathway, sildenafil avoids cGMP degradation and therefore an increase of PKG activation is observed. PKG phosphorylates defective CFTR proteins and corrects their function. Moreover, via cGMP-independent pathway sildenafil activates the exocytotic delivery of CFTR molecules and their insertion into the plasma membrane, increasing their number on it. However, it was observed that is necessary a high concentration of this drug to achieve its beneficial effects so it could provoke severe adverse effects in patients (Leier et al., 2012). Treatment with sildenafil was safe in patients with CF in which was observed a decreased sputum elastase activity (Taylor-Cousar et al., 2015). However, in children with CF, although sildenafil could have anti-inflammatory and benefits in the quality of life and exercise capacities, it decreases lung function. As respiratory failure is one of the most frequent causes of death in CF disease, the administration of sildenafil should be reconsidered (Reisi et al., 2020). For all these observations, since sildenafil has beneficial effects but is not enough safe for the administration, PDE5 inhibitors more sensitive and specific could be a good therapeutical option for the treatment of CF.

In several types of cancer, including lung cancer, the activity of PDE5 is increased and several PDE5 inhibitors, have shown apoptotic and anti-proliferative effects. They potentiate the effect of other drugs and also have immunological effects since they enhance the immune response (Pantziarka et al., 2018; Theodore et al., 2018). In lung cancer cells, PDE5 inhibitors modulate the endocytosis probably via the increase in cGMP levels and consequently the PKG activity, enhancing the cytotoxic activity of the anti-tumoral drugs doxorubicin and cisplatin. Furthermore, vardenafil enhanced the accumulation and anti-tumoral effect of trastuzumab *in vivo* in a mouse model of lung cancer (Li and Shu, 2014). Sildenafil also increased the anti-proliferative effect of carboplatin in H1048 SCLC cell line and A549 cell line (Domvri et al., 2017) and enhanced de anti-tumor effects of pemetrexed (Booth et al., 2016). According to the benefits observed as potentiators of chemotherapy, PDE5 inhibitors could be a good therapeutical option as chemoadjuvants on the treatment of lung cancer.

### sGC Modulators: Stimulators and Activators

Due to the drawbacks mentioned above, other types of drugs that modulate the activity of sGC and increase cGMP, independently of NO are a potential treatment: stimulators and activators of sGC. Stimulators of the sGC enzyme bind to the enzyme increasing the formation of cGMP. These compounds are independent of NO but require the heme group of sGC to be in a reduced state (Fe^+2^). Activators of sGC activate the enzyme and the formation of cGMP independently of NO and even when the heme group of sGC is in an oxidized state (Fe^+3^) or even when it has been lost (Sandner et al., 2019). These sGC modulator drugs, alone and in combination with PDE5 inhibitors, are promising treatments for lung diseases.

#### sGC Stimulators

Stimulators of sGC have a dual action. On the one hand, they stimulate the enzyme in its native form, independently of NO. On the other hand, they stabilize the binding of NO to the sGC, thereby sensitizing the enzyme to low concentrations of NO (Sandner et al., 2018, 2019). This class of drugs binds to the HNOX domain of the β subunit of the enzyme near the heme group. In this way, they prevent NO from being released from the binding site, by generating a conformational change and thus increasing the catalytic activity of the enzyme (Montfort et al., 2017; Wales et al., 2018).

In animal models of COPD in mice and guinea pigs exposed to tobacco smoke, BAY 41-2272 and riociguat prevented pulmonary emphysema and remodeling. Stimulation of sGC was able to revert the apoptosis induced in endothelial and alveolar epithelial cells after peroxynitrite exposure. Furthermore, sGC stimulators reversed the peroxynitrite-induced down-regulation of the antioxidant enzyme Sod1 and the Fgf10 lung maintenance mediator in airway epithelial cells (Weissmann et al., 2014). These data are in line with other obtained in guinea pigs exposed to tobacco smoke, in which treatment with BAY 41-2272 partially reversed emphysema. Furthermore, a decrease in lung inflammation and ROS after sGC stimulation was observed (Paul et al., 2019). Similar results were obtained in mice models of hypertension and emphysema after treatment with riociguat. Riociguat not only reversed hypertension but also partially reversed emphysema by acting in different pathways. On the one hand, riociguat reverted the increased expression of lung iNOS after tobacco exposure. On the other hand, it was observed in airway epithelial cells that riociguat may contribute to lung regeneration by attenuating the upregulation of the activity of MMPs and by reverting the reduction of proliferation induced by cigarette smoke (Pichl et al., 2019).

In rats, it was observed that the BAY 41-2272 stimulator in synergy with NO relaxes the tracheal muscle, an important effect for the regulation of hyperresponsiveness of the airways that occurs in diseases such as asthma (Toque et al., 2010). A bronchodilator effect was also observed in human lung sections (Koziol-White et al., 2020) and in mouse asthma models, in which the BAY 41-2272 stimulator was able to reverse the hyperresponsiveness of the airways of mice with allergic asthma and restore their lung function (Ghosh et al., 2016).

The BAY 41-2272 stimulator shows an antifibrotic effect in human fibroblasts treated with TGF-β (Beyer et al., 2015; Lambers et al., 2019). However, further investigation is required about the role of stimulators of sGC on the EMT observed in asthma and COPD patients. Riociguat and other stimulators are able to reduce EMT and show antifibrotic effects in several fibrotic diseases in which TGF-β is an important mediator (Hu et al., 2017; Sravani et al., 2020). If similar findings could be demonstrated in lung epithelium, sGC stimulators could be a promising therapeutical option since they might have several effects: reducing fibrosis and lung damage and promoting airway relaxation.

In CF patients, sGC stimulators have a correct action of CFTR function and expression since they increase cGMP levels (Quon and Rowe, 2016). Indeed, riociguat was tested in F508del-homozygous patients in a phase II trial (NCT02170025) that was terminated in 2018. However, although no safety concerns were identified, the clinical development of this drug has not continued.

The stimulator YC-1 has shown inhibition of pathways essential for cancer viability in several types of cancer and it might be used as an anti-tumoral drug since it facilitates apoptosis (Wu et al., 2019). In NSCLC, YC-1 had not significant effect on growth inhibition. However, it had the ability to sensitize cancer cells with primary or acquired resistance to gefitinib treatment. Further investigation is necessary but the combination of gefitinib in patients with sGC stimulators might be a good strategy to overcome the drug resistance in NSCLC (Hu et al., 2020).

#### sGC Activators

Due to the need to search for compounds that activate the sGC enzyme in its oxidized form or without the heme group, the compound BAY 58-2667 (Cinaciguat) was identified. It was the first activator of sGC that in addition to being NO independent, was also heme independent (Stasch et al., 2002). Moreover, there are other activators such as BAY 60-2770, HMR 1766 (Ataciguat), or S-2448, but so far there is not any activator approved for use (Sandner et al., 2019).

After oxidation and inhibition of the sGC enzyme with the ODQ compound, Cinaciguat is capable of activating the sGC enzyme, an effect that is not observed with stimulatory drugs (Stasch et al., 2002). Therefore, these drugs have better pharmacological activity under conditions of oxidative stress where there is an alteration of the redox state of the heme group (Fe^+2^ → Fe^+3^) or even a loss of it, which generates the ubiquitination of sGC (Thoonen et al., 2015). Cinaciguat binds to the cavity of the heme group of sGC, activating the enzyme, stabilizing it, and preventing its degradation (Hoffmann et al., 2009; Meurer et al., 2009; Martin et al., 2010). There is a competition between the heme group and Cinaciguat, so in the presence of ODQ that oxidizes the heme group and makes its binding more unstable, there is a greater effect of the activator (Schmidt et al., 2004).

In a murine model of COPD was observed that cigarette smoke decreased sGC and cGMP levels in the airway epithelium. Administration of Cinaciguat to these cigarette-exposed mice not only restored the epithelial cGMP levels but also, the sGC protein expression and therefore the NO-sGC-cGMP-PKG pathway attenuating airway hyperresponsiveness (Glynos et al., 2013). Similar findings were obtained with the activator BAY 60-2777 in a murine model of asthma, in which treatment with this drug normalized the expression levels of iNOS and sGC in the lungs triggering an anti-inflammatory effect (Baldissera et al., 2016). Moreover, it has been shown that the activator BAY 60-2770, in the same way as the stimulator BAY 41-2272, has a bronchodilator effect in human lung sections (Koziol-White et al., 2020), an effect that has also been observed in animal models of asthma in mice (Ghosh et al., 2016).

The combination of sildenafil with the activator BAY 58-2667, in the presence of ODQ, suppressed the differentiation of pulmonary fibroblasts to myofibroblasts induced by TGF-β (Dunkern et al., 2007). Such as in the case of sGC stimulators, further investigation is necessary about the role of sGC activators on the EMT also observed in the airway epithelium of chronic lung diseases.

Currently, in CF and lung cancer, results with sGC activators have not been published yet. In both diseases, there is an increase in oxidative stress in the lungs. For that reason, due to the mentioned beneficial effects of cGMP levels increase in the airway epithelium of these patients and the ability of these types of compounds to activate the sGC enzyme in its oxidized form, these drugs might be a promising therapeutical option for both pathologies.

## Concluding Remarks and Future Perspectives

Dysregulation of NO concentration and disruption of NO-sGC-GMPc-PKG pathway have several consequences to the integrity of airway epithelium. Increased NO concentration by dysregulation of iNOS activity induce chronic inflammatory responses and nitration of proteins involved in proliferation, apoptosis, or migration among others, triggering bronchial epithelial tissue injury that leads to various pulmonary diseases such as asthma, COPD, or cancer. Moreover, a lack of NO is also detrimental since it has antimicrobial properties and plays an important role in the immune response. Indeed, in CF patients altered iNOS function contributes to the severity of the disease. For that reason, modulation of the iNOS-NO-sGC-GMPc-PKG pathway might be a good strategy for the treatment of the mentioned pathologies. In fact, several drugs that participate in this pathway are currently being studied in different phases of clinical trials.

In asthma, COPD and CF, NO donors are limited due to the instability of NO and its reaction with other ROS, decreasing the activation of sGC. However, in the treatment of cancer, the use of NO donors as chemoadjuvants or in combination with radiotherapy is in phase II clinical studies. iNOS inhibitors have controversial results in COPD and asthma since they reduce NO concentration but also the activity of sGC. Nevertheless, the iNOS inhibitor L-NMMA in combination with pembrolizumab is in clinical phase I study for the treatment of several cancers, including lung cancer. In asthma and COPD, PDE5 inhibitors increase cGMP levels, but the activity of sGC is impaired so there is not enough increase of cGMP levels. In CF patients, PDE5 inhibitors have shown beneficial results but are not enough safe for their administration. For the treatment of cancer, PDE5 inhibitors have shown good results as chemoadjuvants *in vitro* and in animal models.

Due to some disadvantages of the mentioned drugs and the benefits in the epithelial integrity after increase cGMP levels described in this review, stimulators, and activators of sGC activity might be potential therapeutical options for lung diseases since they increase cGMP levels independently of NO concentration. Especially, due to the oxidative stress present in the lungs of cancer, COPD, asthma, and CF patients, it might be promising the use of sGC activators that can activate the sGC in its oxidized form and stabilize it preventing its ubiquitination.

## Author Contributions

MB, JM, CE, and JC conceived and designed revision, analyzed the data, contributed to the writing of the manuscript, revision and final approval of the manuscript. All authors contributed to the article and approved the submitted version.

## Conflict of Interest

The authors declare that the research was conducted in the absence of any commercial or financial relationships that could be construed as a potential conflict of interest.

## Abbreviations

3-NT: 3-nitrotyrosine
ADMA: asymmetric dimethylarginine
AJC: apical junctional complex
ASL: airway surface liquid
CCL2: chemokine (C-C motif) ligand 2
CF: cystic fibrosis
CFTR: CF Transmembrane conductance regulator
cGMP: cyclic guanosine-3′,5′-monophosphate
COPD: chronic obstructive pulmonary disease
COX-2: cyclooxygenase-2
DAMPs: danger-associated molecular patterns
EMT: epithelial to mesenchymal transition
ENaC: epithelial sodium channel
eNOS or NOS3: endothelial nitric oxide synthase
EPO: eosinophil peroxidase
F_E_NO: fraction of exhaled nitric oxide
FGF: fibroblast growth factor
GSNO: S-nitrosoglutathione
GSNOR: GSNO reductase
GTP: guanosine 5 ′ -triphosphate
IFN- γ: interferon γ
IL: interleukin
iNOS or NOS2: inducible nitric oxide synthase
I κ B: inhibitor of nuclear factor κ B
JAM: junctional adhesion molecule
LPS: lipopolysaccharides
MAPK: mitogen activated protein kinase
MBP: major basic protein
MMPs: metalloproteinases
NF- κ B: nuclear transcription factor κ B
nNOS or NOS1: neuronal nitric oxide synthase
NO: nitric oxide
NOS: nitric oxide synthase
NSCLC: non-small cell lung cancer
PAMPs: pathogen-associated molecular patterns
PARs: protease-activated receptors
PDEs: phosphodiesterases
pGC: particulate guanylyl cyclase
PGE2: prostaglandin E2
PKGs: cGMP-dependent protein kinases
PRRs: pattern-recognition receptors
ROS: reactive oxygen species
SCLC: small cell lung cancer
sGC: soluble guanylyl cyclase
SNO: S-nytrosylation
T2 asthma: Type 2 asthma
Tc cell: cytotoxic T cell
TGF- β: transforming growth factor β
Th cell: T-helper cell
TLRs: toll-like receptors
TNF- α: tumor necrosis factor α
TSLP: thymic stromal lymphopoietin
VEGF: vascular endothelial growth factor
WHO: World Health Organization
ZO: zonula occludens
α-SMA: alpha-smooth muscle actin.
